# Circulating Matrix Metalloproteinase-28 Levels Are Related to GRACE Scores and Short-Term Outcomes in Patients with Acute Myocardial Infarction

**DOI:** 10.1155/2020/9206703

**Published:** 2020-05-25

**Authors:** Ke Zhou, Yuanmin Li, Yawei Xu, Rong Guo

**Affiliations:** ^1^Nanjing Medical University, Nanjing, Jiangsu 211166, China; ^2^Department of Cardiology, Shanghai Tenth People's Hospital, Tongji University School of Medicine, Shanghai 200072, China; ^3^Department of Cardio-Thoracic Surgery, Shanghai Tenth People's Hospital, Tongji University School of Medicine, Shanghai 200072, China

## Abstract

**Objective:**

To investigate the relationship between the level of matrix metalloproteinase-28 (MMP-28) in patients with acute myocardial infarction (AMI) and the global registry of acute coronary events (GRACE) scores as well as their short-term prognosis.

**Methods:**

Two hundred eleven patients with AMI were enrolled, and their basic clinical characteristics were collected for determining the GRACE score. We measured the plasma levels of MMP-28 and other biomarkers in the study population. The association of MMP-28 levels with cardiac events and cardiac deaths occurring within 30 days of discharge was evaluated with multivariable Cox proportional hazard models.

**Results:**

The MMP-28 levels were significantly higher in patients with acute ST-elevation myocardial infarction (STEMI) than in patients with non-ST-elevation myocardial infarction (NSTEMI) (*P* < 0.01). Correlation analysis showed that the level of MMP-28 was positively correlated with the GRACE score in patients with AMI (*R*^2^ = 0.366, *P* < 0.05). Cox multivariate regression results showed that MMP-28 was associated with cardiovascular events during the hospitalization and 30 days after discharge (*P* < 0.01). In addition, Kaplan–Meier analysis showed that cardiac events and deaths were significantly higher in patients with MMP-28 ≥ 1.21 ng/mL (all *P* < 0.01).

**Conclusion:**

There is a correlation between the plasma MMP-28 level and GRACE score in patients with AMI. MMP-28 is also associated with cardiovascular events and cardiovascular deaths during the hospitalization of patients and within 30 days of discharge.

## 1. Introduction

Acute myocardial infarction (AMI) is a common cardiovascular disease [1]. Extracellular matrix is considered a dynamic constantly remodeling structure that plays a pivotal role in myocardial repair [2]. Matrix metalloproteinases (MMPs) play a pivotal role in postmyocardial infarction cardiac remodeling as well as in the development of adverse outcomes [3]. MMPs are a group of zinc ion-dependent proteases that degrade collagen and proteoglycans and play an important role in the development of atherosclerosis [4]. As a new member in the family of MMPs, MMP-28 may directly damage the matrix around cells through proteolysis or may cooperate with other MMPs [5]. It has been reported that MMP-28 participates in the process of myocardial remodeling after myocardial infarction [6], and it has some correlation with the severity of coronary artery disease [7]. However, the role of MMP-28 in acute myocardial infarction and its correlation with coronary artery disease remain to be elucidated.

Therefore, in this study, we investigated the relationship between the level of MMP-28 in AMI patients and the global registry of acute coronary events (GRACE) scores as well as their short-term prognosis. We aim to explore the clinical value of MMP-28 in the risk assessment and prognosis prediction for AMI.

## 2. Materials and Methods

### 2.1. Subjects

In this observational, retrospective, single-center study, we evaluated the prognostic value of plasma MMP-28 levels in patients with AMI. A total of 211 patients with AMI who were hospitalized in our center from January 2018 to December 2018 were selected. The study was observational, and no intervention was given to the patients. The study was reviewed and approved by the ethics committee of the hospital. All the patients enrolled in the study provided informed consent.

### 2.2. Patient Selection

#### 2.2.1. Inclusion Criteria

The patients with the following characteristics and clinical presentations were included in the study: (1) all the subjects met the latest diagnostic criteria for AMI [8, 9]. (2) The patient received emergency coronary intervention for the first time. All patients with AMI were treated with standard percutaneous coronary intervention, and coronary angiography was performed through the radial artery or the femoral artery. After a clear diagnosis, balloon angioplasty or stent placement was performed at the problematic coronary artery. (3) The selected subjects signed the informed consent form.

#### 2.2.2. Exclusion Criteria

The patients with the following characteristics and clinical presentations were excluded from the study: (1) age < 30 years; (2) large-scale pulmonary embolism; (3) infectious diseases; (4) malignant tumors; (5) history of genetic cardiomyopathy; (6) combined pericardial disease, infective endocarditis, etc.; (7) severe liver and kidney dysfunction or coagulopathy; (8) pregnancy; and (9) other conditions that made the patient unsuitable for enrollment, as determined by the investigators.

### 2.3. Methods

For patients with AMI, 5 mL of fasting venous blood was collected within 24 h of the onset of the disease, before cardiac catheterization. In the normal control group, 5 mL of fasting venous blood was collected early in the morning. The blood samples were placed at room temperature for 2 h. After centrifugation at 1,000 × *g*, the supernatant was collected and stored in a −80°C refrigerator. The samples were thawed at room temperature before the experiment and mixed thoroughly before measurement. The plasma levels of low-density lipoprotein cholesterol (LDL-C) were analyzed by using an enzymatic method (Roche Diagnostics); triglycerides were analyzed using an enzymatic-colorimetric assay. Serum N-terminal pro-B-type natriuretic peptide (NT-proBNP), hypersensitive C-reactive protein (hs-CRP), creatinine (Cr), hemoglobin A1c, and fasting blood glucose (FBG) were measured using a routine-automated technique in a laboratory at Shanghai Tenth People's Hospital, China. The hs-cTnT was assayed using the Elecsys troponin T-high sensitive assay (Roche Diagnostics, Penzberg, DE). All the assays were carried out according to the manufacturer's instructions. We also measured the heart rate (HR), systolic blood pressure (SBP), and diastolic blood pressure (DBP) of the patients.

The level of MMP-28 was measured using the enzyme-linked immunosorbent assay (ELISA) kit by ELISA Genie (Dublin, Ireland) as per the manufacturer's instructions.

### 2.4. Grading of Coronary Artery Stenosis

Coronary angiography was performed in all the AMI patients using the radial or femoral approach, and the degree of coronary artery disease was determined by two experienced interventional physicians. Among the coronary arteries, if only one major branch had a degree of stenosis > 50%, it was considered a single-branch disease; if two major branches had a degree of stenosis > 50%, it was considered a two-branch disease; if three or more major branches had a degree of stenosis greater than 50%, it was considered a three-branch disease.

### 2.5. GRACE Score Analysis

Eight indices were collected: age of the patient, the HR, SBP, serum Cr, Killip class at the time of admission, cardiac arrest before hospitalization, ST segment shift on the ECG, and elevated myocardial marker levels. The indices were scored using the GRACE software (http://www.outcome.org/grace).

### 2.6. Evaluation of Heart Function

Images of the standard long-axis section, short-axis section, and apical two-chamber view and four-chamber view were acquired using a Vivid-7 ultrasound system from GE (GE Healthcare, Piscataway, NJ, USA), and the left ventricular ejection fraction (LVEF) was measured using the Simpson's method.

### 2.7. Follow-Up Questions

This study involved telephonic follow-up and/or face-to-face follow-up of all the selected patients for 30 days after discharge. The follow-up questions included cardiovascular deaths and cardiovascular events, including nonfatal recurrent myocardial infarction; nonfatal stroke; rehospitalization due to heart failure, angina pectoris, and arrhythmia; and revascularizations, including PCI and coronary artery bypass graft surgery (CABG).

### 2.8. Statistical Analysis

All statistical analyses were carried out using SPSS version 17 (SPSS, Inc., Chicago, IL, USA). The data are represented as the means ± standard deviation. Student's *t*-test or the Wilcoxon matched-pairs, signed-rank test was used to determine the differences between the groups for Gaussian-distributed data and non-Gaussian-distributed data, respectively. The *χ*^2^ test or the Fisher exact test was used to compare dichotomous data. Linear regression was used for correlation analysis. The risks of cardiac events and cardiac deaths were assessed in a multivariate Cox proportional-hazard model to determine whether MMP-28 levels independently predicted the outcome. Other variables that were significantly associated with the outcome were entered into the model in a step-wise procedure. Event-free survival curves were constructed using the Kaplan–Meier method and compared using the log-rank test.

## 3. Results

### 3.1. Baseline Data of the Patients

The baseline data of all the selected patients are shown in Table 1. The levels of NT-proBNP, hs-CRP, and MMP-28 were significantly higher in patients with acute ST-elevation myocardial infarction (STEMI) than in patients with non-ST-elevation myocardial infarction (NSTEMI) (*P* < 0.01). There were significant differences in HR, SBP, DBP, and LVEF between the two groups (*P* < 0.05, Table 1), whereas no significant difference was noted in other parameters between the two groups (*P* > 0.05, Table 1).

### 3.2. Coronary Angiography Results

All the patients received coronary angiography, which was performed using the Judkins technique. The angiography results were measured visually in combination with quantitative analysis on using a computer to determine the degree of coronary stenosis. The coronary angiography results showed that a 70% reduction in the internal diameter of the coronary arteries was significant. According to the arteries and branches involved in stenosis, the condition was classified into left main branch and single-, double-, and triple-branch diseases, and the residual stenosis, thrombosis, dissection, perforation, calcification, and spasm were evaluated. The interventional surgery was considered successful if the residual stenosis was ≤10%, and there was no surgery-related myocardial infarction or death during the operation. The specific results are presented in Table 2.

### 3.3. GRACE Risk Stratification, Plasma MMP-28 Levels of Patients, and Their Correlation

**Figure 1:**
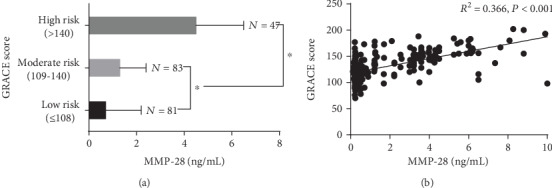
MMP-28 levels in the groups with different risks and the correlation between MMP-28 and GRACE scores. (a) Plasma MMP-28 levels in the groups with different risks. (b) MMP-28 levels were positively correlated with GRACE scores in patients with acute myocardial infarction. ^∗^*P* < 0.05.

**Figure 2:**
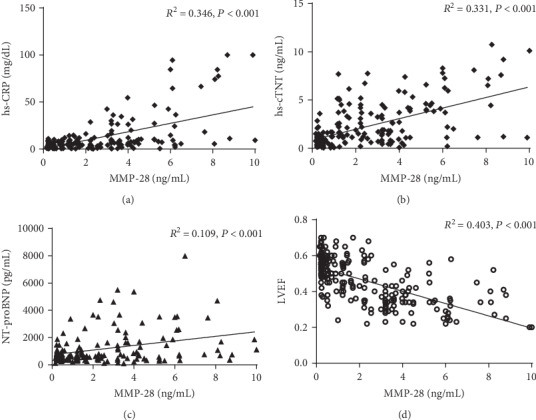
Correlation between the levels of hs-CRP, hs-cTnT, NT-proBNP, LVEF, and MMP-28. Plasma MMP-28 was negatively correlated with hs-CRP (a), hs-cTNT (b), NT-proBNP (c), and LVEF (d) (*P* < 0.001).

The patients were divided into low-risk (*N* = 81), intermediate-risk (*N* = 83), and high-risk (*N* = 47) groups according to the GRACE score (Figure 1(a)), and the MMP-28 levels of the groups were 0.7 ± 1.5, 1.3 ± 1.1, and 4.5 ± 2.0 ng/mL, respectively (Figure 2(a)). Compared with the low-risk and intermediate-risk groups, the high-risk group had a significantly higher MMP-28 level (*P* < 0.05 for both, Figure 1(a)). The difference in the MMP-28 levels between the intermediate-risk and the low-risk groups was also statistically significant (*P* < 0.05, Figure 1(a)).

General linear regression analysis was performed based on the GRACE scores and MMP-28 levels. The results showed that the GRACE score of AMI patients had a significant linear correlation with the plasma MMP-28 level, with an *R*^2^ of 0.366 (*P* < 0.05, Figure 1(b)).

### 3.4. Relationship between Cardiac Function, Plasma MMP-28 Level, and Other Biomarkers

General linear correlation analyses were performed between the plasma MMP-28 level and hs-CRP, hs-cTnT, and NT-proBNP of patients with AMI. The plasma MMP-28 level was found to be positively correlated with hs-CRP, hs-cTnT, and NT-proBNP (*R*^2^ = 0.346, *R*^2^ = 0.331, *R*^2^ = 0.109, *P* < 0.01; Figures 2(a)–2(c)). The MMP-28 levels were found to be negatively correlated with LVEF (*R*^2^ = 0.403, *P* < 0.01; Figure 2(d)).

### 3.5. Determination of the Cut-Off Value

To establish the optimal cut-off value, coronary angiography, combined with hs-cTnT assessment, was used as the confirmatory test for patients with AMI in reference to the plasma MMP-28 levels of 50 healthy individuals. Myocardial infarction was set to be 1, and nonacute myocardial infarction was set to be 0. The ROC curve was drawn where the *y*-axis represented sensitivity and the *x*-axis represented specificity. The AUC_ROC_ was 0.835, and the 95% confidence interval was 0.72–0.91. When 1.21 ng/mL was used as the threshold, the sensitivity and specificity of MMP-28 were relatively higher, with values of 94% and 88%, respectively. Therefore, in the Kaplan–Meier survival analysis, the plasma MMP-28 levels in all the patients were classified with respect to the 1.21 ng/mL cut-off value: ≥1.21 ng/mL group (*N* = 122, male/female = 83/39) and <1.21 ng/mL group (*N* = 89, male/female = 58/31).

### 3.6. Follow-Up Results and Kaplan–Meier Survival Analysis

All the patients in this study completed the follow-up analysis, with an average follow-up period of 24.1 ± 8.3 days. The results of Cox multivariate regression analysis showed that the MMP-28 level was associated with cardiovascular events during the hospitalization and 30 days after discharge (*P* < 0.01, Table 3); NT-proBNP and MMP-28 were closely associated with cardiovascular deaths during the hospitalization and 30 days after discharge (*P* < 0.05, Table 3). The remaining variables appeared to have little correlation with cardiovascular events and cardiovascular deaths.

**Figure 3:**
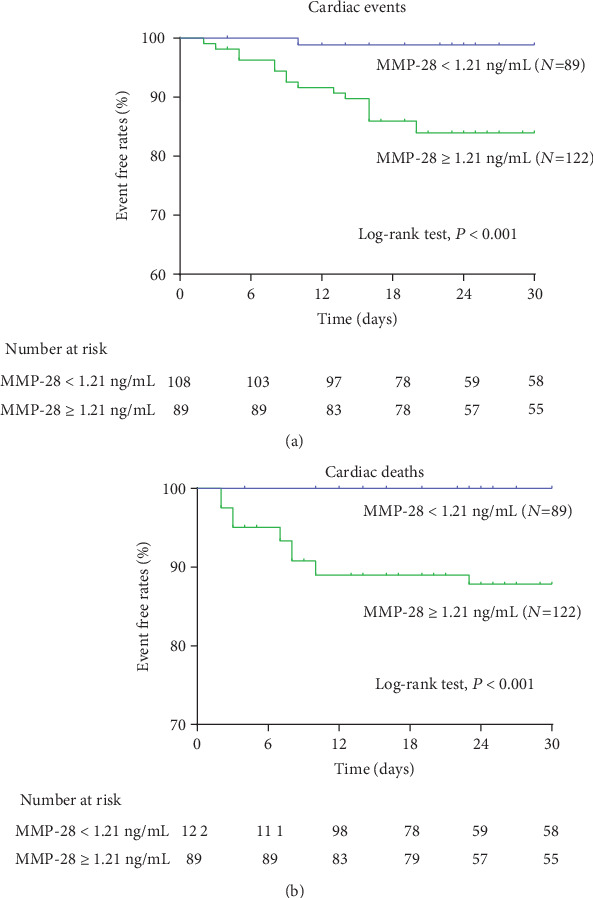
Kaplan–Meier analysis. (a) Kaplan–Meier curve showing the incidence of cardiac events, within 30 days after discharge in patients with acute myocardial infarction. (b) Patients with high MMP-28 levels had significantly higher rates of cardiac death than patients with low MMP-28 levels.

The Kaplan–Meier survival analysis showed that in the MMP-28 ≥ 1.21 ng/mL group, 17 patients had major cardiovascular events and 14 cardiovascular deaths were reported. In the MMP-28 < 1.21 ng/mL group, only one patient had a cardiovascular event, and no cardiovascular death was reported. There was a significant difference between the two groups (*P* < 0.01, Figure 3).

## 4. Discussion

Biomarkers related to AMI have been a topic of interest to cardiologists [10], and in recent years, many reports have focused on the diagnosis, risk stratification, and prognosis of the disease [11]. This study is aimed at exploring the clinical value of MMP-28 in the risk assessment and prognosis prediction of patients with AMI (STEMI and NSTEMI). Our main findings are as follows: (1) MMP-28 is elevated to varying degrees in patients with AMI; (2) plasma MMP-28 level correlates with the GRACE score in patients with AMI; and (3) MMP-28 level correlates with cardiovascular events and cardiovascular deaths during the hospitalization of AMI patients and within 30 days after discharge and can be considered a short-term prognosis predictor for patients with myocardial infarction.

Atherosclerosis is an inflammatory pathological process. MMPs promote the rupture of atherosclerotic plaques by degrading the extracellular matrix. Usually, the occurrence of acute coronary syndrome is caused by the rupture of coronary atherosclerotic plaques, which leads to the thrombosis and occlusion of coronary arteries. The stability of coronary atherosclerotic plaques is affected by the level of collagen [12]. Atherosclerotic plaque instability is the fundamental cause of acute coronary syndromes [13]. Whether plaques are prone to rupture depends mainly on their structure. Unstable plaques are characterized by large lipid cores, thin fiber caps, and are infiltrated by many inflammatory cells, such as monocytes, macrophages, and macrophage-derived foam cells [14]. Therefore, MMPs have been widely studied in the field of atherosclerosis and AMI [15].

MMP-28 (an epithelial protease) is a protein with a molecular mass of 59 kD and is the recently cloned human MMP [16]. It has been reported to play an important role in tumor progression [17–19]. MMP-28 contains a signal peptide sequence, a propeptide domain, a zinc-binding catalytic active site, and a C-terminal heme-binding protein-like domain [5]. MMP-28 is expressed in many normal tissues, such as the testis, small intestine, skin, and lungs, suggesting that it plays a key role in balancing the internal environment of the tissue [20]. Researchers have found that the expression of MMP-28 protein is higher in some tumors than in normal tissues [21]. Studies have shown that the expression of MMP-28 protein is increased in malignant tumors and cancer cell lines [17–19]. However, studies on MMP-28 in the cardiovascular field are limited. Our study provides some clinical evidence and data on the relevance of MMP-28 in cardiovascular diseases.

Ma et al. found that with the increase in age, the expression of MMP-28 in the left ventricle increased by 42% in mouse models. When the MMP-28 gene was knocked out in mice, the levels of inflammatory factors, such as macrophage inflammatory protein- (MIP-) 1*α*, MIP-1*β*, and MMP-9, in the left ventricle increased [22]. These results suggest that MMP-28 is involved in the regulation of myocardial inflammation and extracellular matrix responses. Subsequently, Ma et al. found that knockout of the MMP-28 gene in mice resulted in a more significant ventricular remodeling and deterioration of cardiac function after myocardial infarction. In this study, it was also observed that MMP-28 is closely related to the indices of cardiac function, such as NT-proBNP and LVEF in the population with myocardial infarction [6].

Liu et al. found that the level of MMP-28 increased in patients with stable coronary heart disease and was related to the severity of coronary artery disease, which led to the speculation that MMP-28 might be involved in atherosclerotic lesions [7]. Researchers have also suggested that it can be used as one of the markers in future research [23]. Zhan et al. examined the role of MMP-28 in patients with atrial fibrillation, and the results suggest that MMP-28 is related to the left atrial diameter and the prognosis of heart failure [24]. These studies implicate the value of MMP-28 as a new biomarker for cardiovascular disease from different perspectives. In our study, the level of MMP-28 was significantly elevated in the AMI group, and MMP-28 had a high correlation with hs-cTnT, hs-CRP, NT-proBNP, and LVEF. It is possible that the increased level of MMP-28 in peripheral blood may be one of the markers of plaque rupture or may be involved in the occurrence of AMI. In addition, the Cox risk regression analysis suggests that the level of MMP-28 is an independent risk factor for cardiovascular events and deaths in the short-term after AMI.

Compared with coronary interventional therapy, monitoring of the MMP-28 level in peripheral blood has the following advantages: higher safety, less injury, lower risk, lower cost, and easier to perform. It can be used for early risk assessment and prognosis prediction for AMI, which are helpful in improving the clinical outcome. In addition, with further research, MMP-28 has the potential to be a target for drugs that interfere with plaque stability and can play a role in the early prevention of AMI, the reduction of the incidence of AMI, and targeted treatment.

Finally, there are some limitations to this research. First, this was a single-center observational study, with a relatively small sample size. The results need to be verified with more samples and a larger cohort. In addition, there may be dynamic changes in the MMP-28 levels during the course of the disease, which may be related to the prognosis. Moreover, the follow-up period of subjects in this study was relatively short, and a longer follow-up period may be required.

## 5. Conclusion

There is a correlation between the plasma MMP-28 level and GRACE score in patients with AMI. MMP-28 is associated with cardiovascular events and cardiovascular deaths during the hospitalization of patients and within 30 days after discharge and can be considered a predictor for short-term prognosis in patients with myocardial infarction.

## Figures and Tables

**Table 1:** Baseline characteristics of the included acute myocardial infarction (AMI) patients.

Variables	Control group (*N* = 50)	Disease group		
		STEMI (*N* = 142)	NSTEMI (*N* = 69)	Total (*N* = 211)
Age (yrs)	34.2 ± 5.5	60.0 ± 8.1	59.4 ± 10.4	59.6 ± 9.7
Gender (M/F)	25/25	90/52	51/18	141/70
hs-cTnT (ng/mL)	0.04 ± 0.02	2.14 ± 1.87	1.89 ± 2.06	2.18 ± 2.20
NT-proBNP (pg/mL)	74.2 ± 52.3	1973.8 ± 1639.1^∗^	744.8 ± 516.4^∗^	1146.7 ± 1176.0
hs-CRP (mg/dL)	0.3 ± 0.2	7.5 ± 5.3^∗^	4.4 ± 2.9^∗^	5.4 ± 4.1
MMP-28 (ng/mL)	0.5 ± 0.3	4.3 ± 1.8^∗^	1.4 ± 1.9^∗^	2.4 ± 2.3
BMI (kg/m^2^)	23.3 ± 2.4	24.1 ± 2.1	24.2 ± 2.7	24.2 ± 2.5
Blood glucose profiles				
FBG (mmol/L)	5.4 ± 0.6	6.1 ± 1.2	6.3 ± 1.3	6.2 ± 1.3
HbA1C (%)	5.4 ± 0.6	6.3 ± 0.7	6.4 ± 0.7	6.4 ± 0.7
Lipid profiles				
TC (mmol/L)	4.8 ± 0.6	5.1 ± 0.7	5.0 ± 0.8	5.1 ± 0.7
LDL-C (mmol/L)	3.0 ± 0.5	3.0 ± 0.9	3.2 ± 1.0	3.1 ± 1.0
Heart rate (bpm)	77.6 ± 5.8	81.2 ± 16.2	76.3 ± 12.9	77.9 ± 14.2
SBP (mmHg)	128.4 ± 11.7	126.1 ± 22.9	135.5 ± 20.4	132.4 ± 21.6
DBP (mmHg)	64.1 ± 4.7	60.8 ± 10.9	73.6 ± 10.5	72.1 ± 10.9
LVEF (%)	66.5 ± 3.4	33.4 ± 7.4	51.6 ± 9.9	45.7 ± 12.5
eGFR (mL/min/1.73 m^2^)	92.3 ± 4.5	84.5 ± 9.8	87.2 ± 11.8	86.4 ± 11.2

hs-cTnT: hypersensitive cardiac troponin T; NT-proBNP: N-terminal pro-B-type natriuretic peptide; hs-CRP: hypersensitive C-reactive protein; BMI: body mass index; FBG: fasting blood glucose; HbA1C: hemoglobin A1c; TC: total cholesterol; LDL-C: low-density lipoprotein-cholesterol; SBP: systolic blood pressure; DBP: diastolic blood pressure; LVEF: left ventricular eject fraction; eGFR: estimated glomerular filtration rate. ^∗^Significant differences between two groups; *P* < 0.05.

**Table 2:** Coronary intervention data of the patients with acute myocardial infarction (AMI; *N* = 211).

Data on coronary intervention	*N* (%)
Coronary angiography performed	205 (97.2)
Time between admission and angiography (hours)	
≤24	155 (73.5)
>24–48	43 (20.4)
>48	7 (3.3)
Number of vessel disease	
0	0 (0)
1	88 (41.7)
2	52 (24.6)
3	38 (18.0)
LM	12 (5.7)
LM+3	15 (7.1)
Treatment	
Conservative	10 (4.7)
PTCA/PCI	192 (90.9)
CABG	3 (1.4)

PTCA: percutaneous transluminal coronary angioplasty; LM: left main; PCI: percutaneous coronary intervention; CABG: coronary artery bypass grafting.

**Table 3:** Results of the multivariate Cox proportional hazard analysis.

Outcome/variables	Hazard ratio	95% CI	*P* value
Cardiac events			
Age (yrs)	1.02	0.97–1.08	0.39
Gender (M/F)	1.59	0.53–4.71	0.41
BMI (kg/m^2^)	1.16	0.97–1.39	0.10
NT-proBNP (pg/mL)	1.00	1.00–1.00	0.92
hs-CRP (mg/dL)	0.95	0.88–1.01	0.14
hs-cTNT (ng/mL)	0.81	0.63–1.06	0.12
eGFR (mL/min/1.73 m^2^)	1.02	0.97–1.07	0.48
Grace scores	1.01	0.99–1.04	0.34
LVEF (%)	0.06	0.00–32.59	0.37
MMP-28 (ng/mL)	1.64	1.14–2.36	0.008^∗∗^
In-hospital and 30-day cardiac mortality			
Age (yrs)	1.02	0.94–1.11	0.67
Gender (M/F)	1.05	0.22–1.23	0.95
BMI (kg/m^2^)	0.85	0.59–1.23	0.39
NT-proBNP (pg/mL)	1.00	1.00–1.00	0.009^∗∗^
hs-CRP (mg/dL)	1.00	0.98–1.03	0.98
hs-cTNT (ng/mL)	0.79	0.59–1.04	0.09
eGFR (mL/min/1.73 m^2^)	1.07	0.99–1.15	0.08
Grace scores	1.05	0.98–1.11	0.14
LVEF (%)	3.50	0.00–4997.72	0.74
MMP-28 (ng/mL)	1.85	1.08–3.16	0.02^∗^

95% CI: 95% confidence interval; ^∗^*P* < 0.05; ^∗∗^*P* < 0.01.

## Data Availability

All data generated or analyzed during this study are included in this published article.
